# Collapsing glomerulopathy associated with parvovirus B19 and systemic lupus erythematosus in a patient with *APOL1* high-risk variant for nephropathy

**DOI:** 10.1590/2175-8239-JBN-2024-0104en

**Published:** 2025-01-10

**Authors:** Thaíza Passaglia Bernardes, Thalita Alvarenga Ferradosa Paula, Gabriel Teixeira Montezuma Sales, Patrícia Varela Calais, Renato Demarchi Foresto, Luiz Antonio Moura, Marcelino de Souza Durão, João Bosco Pesquero, Gianna Mastroianni Kirsztajn

**Affiliations:** 1 Universidade Federal de São Paulo, Departamento de Medicina, São Paulo, SP, Brazil.; 2 Universidade Federal de São Paulo, Departamento de Biofísica, Centro de Pesquisa e Diagnóstico em Doenças Genéticas, São Paulo, SP, Brazil.; 3 Fundação Oswaldo Ramos, Hospital do Rim, São Paulo, SP, Brazil.

**Keywords:** Collapsing Glomerulopathy, Parvovirus B19, Systemic Lupus Erythe­matosus, APOL1, Nephropathy

## Abstract

Collapsing glomerulopathy (CG) has a severe course typically associated with viral infections, especially HIV and parvovirus B19, systemic lupus erythematosus (SLE), among other etiologies. A 35-year-old woman with recent use of a JAK inhibitor due to rheumatoid arthritis presented with a 2-week history of fever, cervical adenopathy, and facial erythema. After admission, anemia, hypoalbuminemia, proteinuria, and severe acute kidney injury were noted. SLE was diagnosed and parvovirus B19 DNA was detected in serum samples. Kidney biopsy showed CG without any typical features of lupus nephritis. The patient was treated with prednisone and presented marked improvement of anemia and kidney function after a few weeks. In this case, the patient with SLE presented CG possibly caused by parvovirus B19 infection associated with homozygous apolipoprotein 1 (*APOL1*) G1 genotype, which has been described as a determinant risk factor for this glomerulopathy. It is not clear whether SLE had a causal relationship with glomerular disease or was a concurrent cause. Treatment can be challenging in such a context, as no antiviral drug is efficient and immunosuppression has no discernable benefit, although steroid use was efficient in treating renal manifestations in this case.

## Introduction

Focal and segmental glomerulosclerosis (FSGS) is the most common primary glomerulopathy in Brazil and is divided into 5 subtypes (Tip Lesion, Collapsing, Perihilar, Cellular, and Not Otherwise Specified) according to the Columbia classification^1,2^. Collapsing glomerulopathy (CG) is the most severe variant, characterized by immunosuppression-resistant nephrotic syndrome and progression to end-stage renal disease (ESRD) in months to a few years^3^.

Glomerular findings of CG include collapse of capillary loops, hyperplasia of podocytes, and tubular microcysts, which have been described primarily as a spectrum of FSGS, but recently also in other glomerulopathies such as IgA nephropathy^4^. Disease mechanism is not completely understood, but genetic susceptibility and an environmental second hit are complementary. A strong association has been extensively shown between collapse and homozygous *APOL1* gene risk variants (G1 and G2) in patients with HIV and parvovirus B19, systemic lupus erythematosus (SLE), and some drugs such as pamidronate and interferon^5,6,7^.

The current classification of lupus nephritis does not include podocytopathies, but cases associated with CG and minimal change disease (MCD) have been described, and a probable causal relationship has been accepted. The presence of mesangial immune complex deposits and endocapillary proliferation reinforce this theory, as they represent morphological patterns compatible with lupus flare^7,8^. However, lupus patients are also susceptible to other possible etiologies, especially viral infections, as they usually use immunosuppressors.

Hereby we present a case of CG in a SLE patient with homozygous *APOL1* high-risk variant for nephropathy and diagnosis of acute parvovirus infection.

## Case Report

A 35-year-old black woman was admitted to our hospital complaining of a 2-week fever, nocturnal sweating, facial erythema, and painful oral ulcers. She denied articularRelato de Caso or respiratory symptoms. Physical examination showed normal vital signs, paleness, tender cervical adenopathy, and no hepatosplenomegaly.

Eighteen months before, she had been diagnosed with rheumatoid arthritis by another medical service when presenting with asymmetric polyarthritis of hands, ankles, and knees associated with morning stiffness of 1 hour. Prednisone and methotrexate were started, but the latter was interrupted a few months later due to nausea and vomiting, and tofacitinib (JAK inhibitor) was then prescribed. Tofacitinib was continued for around 6 months and discontinued 2 months before admission due to financial issues. After that she was maintained on low-dose prednisone.

Laboratory exams collected at admission revealed serum creatinine (Cr) of 3.74 mg/dL, urea 108 mg/dL, hemoglobin (Hb) 10.9 g/dL, leukocytes 2,620/mm^3^, platelets 137,000/mm^3^, total bilirubin 0.21 mg/dL, AST 191 U/L, ALT 110 U/L, ferritin 5,628 ng/mL, DHL 777 U/L, albumin 1.9 g/dL, and normal complement levels. A baseline serum creatinine was not available. Urinalysis showed 222,000 leukocytes/mL, 110,000 red blood cells/mL, and 10.3 g/L protein. Additional tests performed during the investigation showed negative toxoplasmosis IgG and IgM, HIV, HTLV, syphilis, hepatitis B and C; homogeneous pattern on ANA > 1:1280, anti-dsDNA > 1:320, and negative anti-ENA and rheumatoid factor. Ultrasound showed normal-sized kidneys with diffuse and increased echogenicity. The typical autoantibodies panel with a compatible clinical presentation supported the SLE diagnosis. As a patient that had never had a documented renal flare and with atypical signs of lupus activity as adenopathy, painful oral ulcers, and high liver enzymes, it was decided to investigate other causes of acute lymph node enlargement and glomerulopathy while initiating a 3-day 1 g intravenous methylprednisolone treatment.

**Figure 1:**
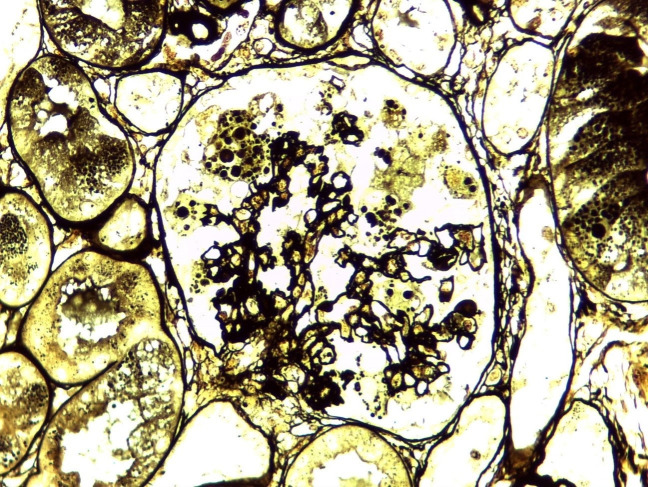
Light microscopy (x 400) – Jones staining – showing a glomerulus with segmental collapsed capillary walls surrounded by hypertrophic/degenerative podocytes, with hyaline granules next to a dilated tubule filled with a cylinder.

**Figure 2:**
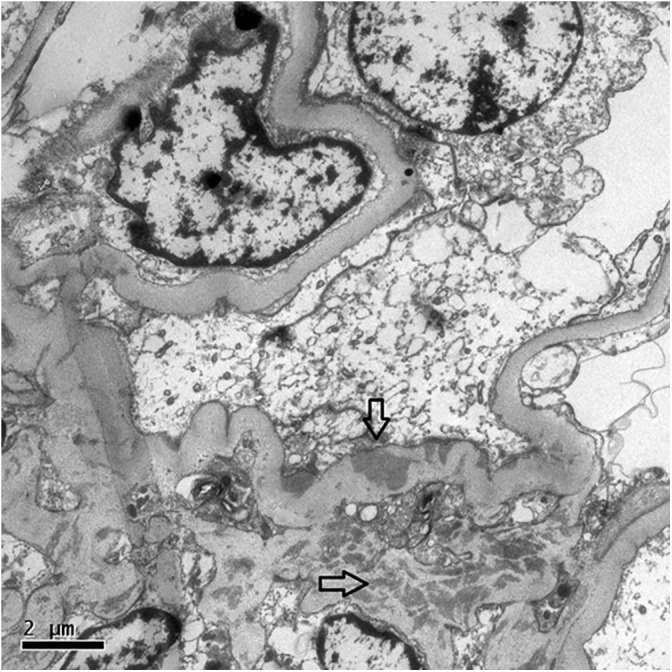
Transmission electron microscopy (x 8000) showing capillary walls with retraction and tortuosity. Electron dense deposits within the mesangium (lower arrow) and a few subepithelial deposits in the capillary wall (upper arrow).

In the following days, she presented progressive anemia (Hb 9.0 g/dL on day 2 and 7.7 g/dL on day 7) and progressive renal failure. Intermittent hemodialysis was initiated on day 6, and a kidney biopsy was performed. It showed 11 glomeruli, all of them with degenerated and hypertrophic podocytes. One glomerulus presented with a segmental collapse of capillary walls. Renal interstitium showed dilated tubules filled with hyaline casts, mild fibrosis, and tubular atrophy (Figure 1). Immunofluorescence microscopy showed segmental and irregular IgM (++/4+) and C3 (traces) deposits. By electron microscopy, mild mesangial and sparse subepithelial deposits were seen associated with capillary wall retraction and tortuosity (Figure 2).

Complementary investigation of viral agents resulted in negative EBV serology, positive CMV IgG, and negative CMV IgM serology, and serum polymerase chain reaction (PCR) for CMV. However, IgM and IgG serology, as well as serum PCR for parvovirus B19 were positive. *APOL1* gene sequencing was performed, revealing a homozygous G1 genotype.

Prednisone 1 mg/kg (70 mg/day) was initiated with clinical and laboratory improvement in the following days. The last dialysis was performed on day 8 after hospital admission, and Cr decreased since then (4.2, 3.1, and 2.4 mg/dL, respectively on days 10, 13, and 15). Spot urine protein/creatinine ratio (UPCR) on day 9 was 2.31 g/g. After discharge, in her first outpatient visit (day 18 after admission), she presented a Cr of 1.09 mg/dL. A prednisone tapering was initiated, and after 3 months, the dose was 30 mg/day. Laboratory exams then showed Cr 0.69 mg/dL, albumin 4.3 g/dL, hemoglobin 16.2 g/dL, UPCR 1.24 g/g, and a 24h-urinary protein of 0.93 g.

## Discussion

The present case is unique because it presented two possible etiologies that justify clinical and histologic findings along with a nephropathy high-risk *APOL1* genotype. Anti-dsDNA, hypocomplementemia, oral ulcers, and anemia are hallmarks of lupus activity. Nonetheless, the last three can also be found in acute parvovirus B19 infection. Painful adenopathy and fever can be easily attributed to viral disease but are also possible features of autoimmune entities. Due to severe kidney injury, a high dose of steroids was initially indicated. There was a striking improvement in the clinical picture, but prednisone tapering was initiated after a month considering the positivity of viral tests. The satisfactory course of the disease suggests the coexistence of immunological mechanisms along with direct viral injury from podocyte infection. It is of note that Besse et al*.* ^7^ has reported a case of parvovirus B19 infection associated with CG and *APOL1* high-risk genotype, where the patient was treated with human intravenous immunoglobulin (IVIG) and 2 weeks of 1 mg/kg methylprednisolone, but with no satisfactory clinical response^6^.

Parvovirus B19 is a DNA virus discovered in 1975, which causes, especially in childhood and immunosuppressed patients, different clinical presentations, ranging from no symptoms to aplastic anemia. Since the beginning of this century, many case reports have described glomerular diseases related to this virus, with a variety of histological patterns (from endocapillary and mesangial proliferation to CG)^7,9,10,11^. Viral DNA isolation (by PCR and in situ hybridization) has been described in podocytes and tubular cells, especially in CG cases. This suggests viral direct injury as a possible mechanism of glomerular disease. Besides that, deposition of immune complexes is commonly present and can also play a role, but this finding is not consistent in the scientific literature^11^. Prognosis is heterogeneous, as some authors have described progression to ESRD and others have reported spontaneous remission. No antiviral is known to be efficient and anecdotal use of IVIG has been described in severe cases. Support therapy is currently recommended as a cornerstone^10,12^.

Cases of CG induced by drugs have been documented, primarily related to interferon and pamidronate. Our patient had used tofacitinib, which has not been associated with glomerulopathies. However, an increased risk of viral infections after its use was demonstrated in clinical trials, especially by the varicella-zoster virus^13,14^. The drug impairs interferon signaling by inhibiting the JAK pathway, causing inhibition of lymphocyte proliferation and, consequently, inhibition of the immune response against viral infections^14,15^.

Also, Salvatore et al.^16^ in 2012 reported a cohort of 19 patients with SLE and CG, suggesting an association. Most of them presented clinical signs of lupus activity at biopsy, but only around 50% had classical histologic features, predominantly the mesangial proliferative pattern. Other case reports were previously published with similar findings, including rapid progression to ESRD^17,18,19^. Possible mechanisms of podocyte injury involve T cells and an antibody-mediated process in the context of a genetically susceptible population^8^. The response rate to steroids in patients with SLE and CG is highly variable, but Salvatore et al.^16^ showed that 6 out of 13 patients did not progress to ESRD during a median follow up period of 2 years. The number of collapsing lesions and proportion of global glomerulosclerosis and tubular atrophy were correlated with response. Our patient presented little evidence of an immunocomplex lesion by light microscopy, while electron microscopy showed some mesangial and subepithelial electron-dense deposits, which can be attributed to lupus, but not as a mechanism of a severe podocyte lesion. Although proliferative lupus nephritis always demands induction and maintenance treatment, lupus podocytopathies can be treated exactly as primary FSGS, especially in cases where another reversible etiology was identified.

The G1 and G2 variants of *APOL1* are strongly associated with the risk of developing CG, either idiopathic or secondary to exposure to triggers such as infections (HIV, HCV, parvovirus) or autoimmunity. The mechanisms to explain how these haplotypes make podocytes susceptible to different stressors have been investigated, with reports suggesting the hypothesis of loss-of-function mutations, which explains why the presence of two high-risk variants is needed to increase glomerulopathy risk^20^. Another possible explanation is the two-hit theory, in which genetic susceptibility combined with increased endogenous production of Th1 cytokines (especially interferon) in response to viruses or autoimmunity may stimulate the production of a cell-toxic *APOL1* variant protein that leads to increased podocyte injury^5,6,7^.

This case shows that patients with CG can have concurrent etiologies for this glomerulopathy and need a thorough investigation, even when there is already an etiology that may justify glomerular presentation, especially when atypical features are present. Our patient presented significant improvement with immunosuppression, in contrast to most cases of CG, suggesting that steroids may have a role in treatment even when a viral etiology is present, cautiously considering the clinical picture of each patient.
