# Supplementary material for: Collapsing glomerulopathy associated with parvovirus B19 and systemic lupus erythematosus in a patient with APOL1 high-risk variant for nephropathy
Source: J Bras Nefrol. 2025 Jan 10;47(1):e20240104. doi: 10.1590/2175-8239-JBN-2024-0104en (PMC11723606; doi:10.1590/2175-8239-JBN-2024-0104en)
Supplement: Translation

# Translation: Glomerulopatia colapsante associada a parvovírus B19 e lúpus eritematoso sistêmico em paciente com variante *APOL1* de alto risco para nefropatia

**DOI:** 10.1590/2175-8239-JBN-2024-0104pt

**Authors:** Thaíza Passaglia Bernardes, Thalita Alvarenga Ferradosa Paula, Gabriel Teixeira Montezuma Sales, Patrícia Varela Calais, Renato Demarchi Foresto, Luiz Antonio Moura, Marcelino de Souza Durão, João Bosco Pesquero, Gianna Mastroianni Kirsztajn

**Affiliations:** 1 Universidade Federal de São Paulo, Departamento de Medicina, São Paulo, SP, Brasil.; 2 Universidade Federal de São Paulo, Departamento de Biofísica, Centro de Pesquisa e Diagnóstico em Doenças Genéticas, São Paulo, SP, Brasil.; 3 Fundação Oswaldo Ramos, Hospital do Rim, São Paulo, SP, Brasil.

**Keywords:** Glomerulopatia Colapsante, Parvovírus B19, Lúpus Eritematoso Sistêmico, APOL1, Nefropatia

## Abstract

A glomerulopatia colapsante (GC) apresenta um curso grave, tipicamente associado a infecções virais, especialmente HIV e parvovírus B19, lúpus eritematoso sistêmico (LES), entre outras etiologias. Uma mulher de 35 anos, com uso recente de um inibidor de JAK devido à artrite reumatoide, apresentou histórico de duas semanas de febre, adenopatia cervical e eritema facial. Após a admissão, observou-se anemia, hipoalbuminemia, proteinúria e injúria renal aguda grave. Foi diagnosticado LES e o DNA do parvovírus B19 foi detectado em amostras de soro. A biópsia renal revelou GC sem quaisquer características típicas de nefrite lúpica. A paciente foi tratada com prednisona e apresentou melhora acentuada da anemia e da função renal após algumas semanas. Neste caso, a paciente com LES apresentou GC possivelmente causada por infecção por parvovírus B19 associada ao genótipo homozigoto G1 da apolipoproteína 1 (*APOL1*), que tem sido descrito como um fator de risco determinante para essa glomerulopatia. Não está claro se o LES teve uma relação causal com a doença glomerular ou se foi uma causa concomitante. O tratamento pode ser desafiador nesse contexto, uma vez que nenhum medicamento antiviral é eficaz e a imunossupressão não apresenta benefícios perceptíveis, embora o uso de esteroides tenha sido eficaz no tratamento das manifestações renais nesse caso.

## Introdução

A glomeruloesclerose segmentar focal (GESF) é a glomerulopatia primária mais comum no Brasil e está dividida em cinco subtipos (*Tip Lesion*, Colapsante, Perihilar, Celular e Sem Outra Especificação) de acordo com a classificação de Columbia^1,2^. A glomerulopatia colapsante (GC) é a variante mais grave, caracterizada por síndrome nefrótica resistente à imunossupressão e progressão para doença renal em estágio terminal (DRET) no período de meses a alguns anos^3^.

Os achados glomerulares da GC incluem colapso de alças capilares, hiperplasia de podócitos e microcistos tubulares, que foram descritos principalmente como um espectro da GESF, mas também recentemente em outras glomerulopatias, como a nefropatia por IgA^4^. O mecanismo da doença não é totalmente compreendido, mas a suscetibilidade genética e um segundo fator ambiental são complementares. Uma forte associação entre colapso e variantes de risco homozigóticas do gene *APOL1* (G1 e G2) foi amplamente demonstrada em pacientes com HIV e parvovírus B19, lúpus eritematoso sistêmico (LES) e alguns medicamentos, como pamidronato e interferon^5,6,7^.

A classificação atual da nefrite lúpica não inclui podocitopatias, porém foram descritos casos associados à GC e à doença de lesão mínima (DLM), e uma provável relação causal foi aceita. A presença de depósitos de imunocomplexos mesangiais e a proliferação endocapilar reforçam essa teoria, já que representam padrões morfológicos compatíveis com a exacerbação do lúpus^7,8^. No entanto, pacientes com lúpus também estão suscetíveis a outras possíveis etiologias, especialmente infecções virais, visto que geralmente fazem uso de imunossupressores.

Neste artigo, apresentamos um caso de GC em uma paciente com LES e variante *APOL1* homozigótica de alto risco para nefropatia, com o diagnóstico de infecção aguda por parvovírus.

## Relato de Caso

Uma mulher negra de 35 anos deu entrada em nosso hospital com queixa de febre há duas semanas, sudorese noturna, eritema facial e úlceras orais dolorosas. Ela negou sintomas articulares ou respiratórios. O exame físico revelou sinais vitais normais, palidez, adenopatia cervical sensível e ausência de hepatoesplenomegalia.

Dezoito meses antes, ela havia sido diagnosticada com artrite reumatoide por outro serviço médico, ao apresentar poliartrite assimétrica das mãos, tornozelos e joelhos associada à rigidez matinal de uma hora. Iniciou-se o uso de prednisona e metotrexato, porém este último foi interrompido alguns meses depois devido a náuseas e vômitos, sendo então prescrito o tofacitinibe (inibidor de JAK). O tofacitinibe foi administrado por cerca de 6 meses e descontinuado 2 meses antes da hospitalização devido a questões financeiras. Em seguida, ela foi mantida com prednisona em baixa dose.

Os exames laboratoriais coletados na admissão revelaram creatinina sérica (Cr) de 3,74 mg/dL, ureia 108 mg/dL, hemoglobina (Hb) 10,9 g/dL, leucócitos 2.620/mm^3^, plaquetas 137.000/mm^3^, bilirrubina total 0,21 mg/dL, AST 191 U/L, ALT 110 U/L, ferritina 5.628 ng/mL, DHL 777 U/L, albumina 1,9 g/dL e níveis normais de complemento. A creatinina sérica basal não estava disponível. A urinálise revelou 222.000 leucócitos/mL, 110.000 hemácias/mL e 10,3 g/L de proteína. Exames adicionais realizados durante a investigação mostraram IgG e IgM negativos para toxoplasmose, HIV, HTLV, sífilis, hepatite B e C; padrão homogêneo no FAN > 1:1280, anti-dsDNA > 1:320 e anti-ENA e fator reumatoide negativos. A ultrassonografia revelou rins de tamanho normal com ecogenicidade difusa e aumentada. O painel típico de autoanticorpos com uma apresentação clínica compatível corroborou o diagnóstico de LES. Como se tratava de uma paciente que nunca havia tido uma exacerbação renal documentada e com sinais atípicos de atividade lúpica, como adenopatia, úlceras orais dolorosas e enzimas hepáticas elevadas, decidiu-se pela investigação de outras causas de linfonodomegalia aguda e glomerulopatia, ao mesmo tempo em que se iniciou um tratamento de 3 dias com 1 g de metilprednisolona intravenosa.

Nos dias seguintes, a paciente apresentou anemia progressiva (Hb 9,0 g/dL no segundo dia e 7,7 g/dL no sétimo dia) e insuficiência renal progressiva. A hemodiálise intermitente foi iniciada no sexto dia, e uma biópsia renal foi realizada. O exame mostrou 11 glomérulos, todos com podócitos degenerados e hipertróficos. Um glomérulo apresentou colapso segmentar das paredes capilares. O interstício renal mostrou túbulos dilatados preenchidos com cilindros hialinos, fibrose leve e atrofia tubular (Figura 1). A microscopia de imunofluorescência demonstrou depósitos segmentares e irregulares de IgM (++/4+) e C3 (traços). Na microscopia eletrônica, foram observados discretos depósitos mesangiais e depósitos subepiteliais esparsos associados à retração e tortuosidade da parede capilar (Figura 2).

**Figura 1:** Microscopia óptica (x 400) - coloração de Jones - mostrando glomérulo com paredes capilares segmentares colapsadas circundadas por podócitos hipertróficos/degenerativos, com grânulos hialinos ao lado de um túbulo dilatado preenchido por um cilindro.

**Figura 2:** Microscopia eletrônica de transmissão (x 8000) mostrando paredes capilares com retração e tortuosidade. Depósitos elétron-densos no mesângio (seta inferior) e alguns depósitos subepiteliais na parede capilar (seta superior).

A investigação complementar de agentes virais resultou em sorologia negativa para EBV, CMV IgG positivo e sorologia CMV IgM e reação em cadeia da polimerase (PCR) sérica para CMV negativos. No entanto, a sorologia de IgM e IgG, bem como a PCR sérica para parvovírus B19, foram positivas. Realizou-se o sequenciamento do gene *APOL1*, revelando um genótipo G1 homozigoto.

Foi iniciado o tratamento com prednisona 1 mg/kg (70 mg/dia), com melhora clínica e laboratorial nos dias seguintes. A última diálise foi realizada no oitavo dia após a admissão hospitalar, e a Cr apresentou redução desde então (4,2; 3,1 e 2,4 mg/dL, respectivamente nos dias 10, 13 e 15). A relação proteína/creatinina urinária (RPCU) em amostra isolada de urina no 9º dia foi de 2,31 g/g. Após a alta, em sua primeira consulta ambulatorial (18 dias após a internação), ela apresentou Cr de 1,09 mg/dL. Foi iniciada uma redução gradual da prednisona e, após 3 meses, a dose era de 30 mg/dia. Na sequência, os exames laboratoriais revelaram Cr 0,69 mg/dL, albumina 4,3 g/dL, hemoglobina 16,2 g/dL, RPCU 1,24 g/g e proteína urinária de 24 horas de 0,93 g.

## Discussão

O presente caso é único porque apresentou duas possíveis etiologias que justificam os achados clínicos e histológicos, juntamente com um genótipo de alto risco do *APOL1* para nefropatia. Anti-DsDNA, hipocomplementemia, úlceras orais e anemia são marcadores característicos da atividade do lúpus. No entanto, os três últimos também podem ser encontrados na infecção aguda por parvovírus B19. Adenopatia dolorosa e febre podem ser facilmente atribuídas a doenças virais, mas também são possíveis características de entidades autoimunes. Devido à injúria renal grave, indicou-se inicialmente uma alta dose de esteroides. Houve uma melhora notável no quadro clínico, mas a redução gradual da prednisona foi iniciada após um mês, considerando a positividade dos testes virais. O curso satisfatório da doença sugere a coexistência de mecanismos imunológicos juntamente com a injúria viral direta decorrente da infecção dos podócitos. Cabe ressaltar que Besse et al*.* ^7^ relataram um caso de infecção por parvovírus B19 associada à GC e ao genótipo de alto risco do *APOL1*, em que o paciente foi tratado com imunoglobulina humana intravenosa (IGIV) e 2 semanas de 1 mg/kg de metilprednisolona, mas sem resposta clínica satisfatória^6^.

O parvovírus B19 é um vírus de DNA descoberto em 1975, que causa, especialmente na infância e em pacientes imunossuprimidos, diferentes apresentações clínicas, que vão desde a ausência de sintomas até a anemia aplástica. Desde o início deste século, muitos relatos de casos têm descrito doenças glomerulares relacionadas a esse vírus, com uma variedade de padrões histológicos (de proliferação endocapilar e mesangial a GC)^7,9,10,11^. O isolamento do DNA viral (por PCR e hibridização *in situ*) foi descrito em podócitos e células tubulares, especialmente em casos de GC. Isso sugere a lesão viral direta como um possível mecanismo de doença glomerular. Além disso, a deposição de complexos imunes é comumente observada e também pode desempenhar um papel, mas esse achado não é consistente na literatura científica^11^. O prognóstico é heterogêneo, visto que alguns autores descreveram progressão para DRET e outros relataram remissão espontânea. Não se tem conhecimento de nenhum antiviral que seja eficaz, e o uso anedótico de IGIV foi descrito em casos graves. Atualmente, a terapia de suporte é recomendada como a base do tratamento^10,12^.

Casos de GC induzidos por medicamentos têm sido documentados, principalmente relacionados ao interferon e ao pamidronato. Nossa paciente havia feito uso de tofacitinibe, que não foi associado a glomerulopatias. Entretanto, um risco maior de infecções virais após seu uso foi demonstrado em estudos clínicos, especialmente pelo vírus varicela-zoster^13,14^. O medicamento prejudica a sinalização do interferon ao inibir a via JAK, causando inibição da proliferação de linfócitos e, consequentemente, da resposta imune contra infecções virais^14,15^.

Além disso, Salvatore et al.^16^, em 2012, relataram uma coorte de 19 pacientes com LES e GC, sugerindo uma associação. A maioria deles apresentou sinais clínicos de atividade lúpica na biópsia, mas somente cerca de 50% tinham características histológicas clássicas, predominantemente o padrão proliferativo mesangial. Outros relatos de casos foram publicados anteriormente com achados semelhantes, incluindo a rápida progressão para DRET^17,18,19^. Os possíveis mecanismos de lesão dos podócitos envolvem células T e um processo mediado por anticorpos no contexto de uma população geneticamente suscetível^8^. A taxa de resposta aos esteroides em pacientes com LES e GC é altamente variável, mas Salvatore et al.^16^ mostraram que 6 de 13 pacientes não progrediram para DRET durante um período médio de acompanhamento de 2 anos. O número de lesões colapsantes e a proporção de glomeruloesclerose global e atrofia tubular foram correlacionados com a resposta. Nossa paciente apresentou poucas evidências de lesão por imunocomplexos à microscopia óptica, enquanto a microscopia eletrônica mostrou alguns depósitos mesangiais e subepiteliais elétron-densos, que podem ser atribuídos ao lúpus, mas não como um mecanismo de lesão grave de podócitos. Embora a nefrite lúpica proliferativa sempre exija tratamento de indução e manutenção, as podocitopatias lúpicas podem ser tratadas exatamente como a GESF primária, especialmente em casos nos quais outra etiologia reversível foi identificada.

As variantes G1 e G2 do *APOL1* estão fortemente associadas ao risco de desenvolver GC, seja idiopática ou secundária à exposição a fatores desencadeantes, como infecções (HIV, HCV, parvovírus) ou autoimunidade. Os mecanismos para explicar como esses haplótipos tornam os podócitos suscetíveis a diferentes estressores têm sido investigados, com relatos sugerindo a hipótese de mutações de perda de função, o que explica por que a presença de duas variantes de alto risco é necessária para aumentar o risco de glomerulopatia^20^. Outra possível explicação é a teoria dos dois golpes, na qual a suscetibilidade genética combinada com o aumento da produção endógena de citocinas Th1 (especialmente interferon) em resposta a vírus ou autoimunidade pode estimular a produção de uma variante da proteína *APOL1* tóxica para as células, levando a um aumento da lesão de podócitos^5,6,7^.

Este caso demonstra que pacientes com GC podem apresentar etiologias concomitantes para essa glomerulopatia e necessitam de uma investigação detalhada, mesmo quando já existe uma etiologia que possa justificar a apresentação glomerular, especialmente quando características atípicas estão presentes. Nossa paciente apresentou uma melhora significativa com a imunossupressão, em contraste com a maioria dos casos de GC, sugerindo que os esteroides podem ter um papel no tratamento mesmo quando uma etiologia viral está presente, considerando com cautela o quadro clínico de cada indivíduo.
